# Variations in Out-of-Hospital Cardiac Arrest Resuscitation Performance and Outcomes in Ohio

**DOI:** 10.5811/westjem.19422

**Published:** 2025-03-15

**Authors:** Michelle M.J. Nassal, Henry E. Wang, Jonathan R. Powell, Justin L. Benoit, Ashish R. Panchal

**Affiliations:** * The Ohio State University Wexner Medical Center, Department of Emergency Medicine, Columbus, Ohio; † University of Cincinnati College of Medicine, Department of Emergency Medicine, Cincinnati, Ohio

## Abstract

**Introduction:**

Understanding characteristics of top-performing emergency medical service (EMS) agencies and hospitals can be an important tool for improving community out-of-hospital cardiac arrest (OHCA) care. We compared deidentified EMS and hospital-level variations in OHCA performance and outcomes in Ohio.

**Methods:**

We analyzed adult OHCA data from the 2019 Ohio Cardiac Arrest Registry to Enhance Survival (Ohio CARES). We limited the analysis to EMS agencies and receiving hospitals with ≥10 OHCA episodes. The primary outcomes were return of spontaneous circulation (ROSC) and survival to hospital discharge. We compared OHCA outcomes between EMS agencies using linear mixed models, with EMS agency as a random effect and adjusting for Utstein variables. We repeated the analysis by receiving hospital. We compared EMS agency population demographics, response times, and resuscitation characteristics of the top 10% of agencies against remaining agencies using chi-squared tests.

**Results:**

We included 2,841 OHCA among 44 EMS agencies in our analysis. The ROSC varied three-fold; mean 27.9%, range 15.8%‒51.0%. Among 40 hospitals, survival varied two-fold; mean 12.9%, range 8.1%‒19.0%. Top-performing EMS agencies included both medium- and large-sized agencies that tended to treat younger patients (59 vs 62 years, P<0.01) in public areas (15.7% vs 12.3%, P<0.01). There were no differences in bystander-witnessed arrest, bystander cardio-pulmonary resuscitation (CPR), or EMS response time. However, top-performing EMS agencies used less mechanical CPR (61.7% vs 76.0%, P<0.01) and were more successful in advanced airway placement (89.6% vs 74.8% P<0.01).

**Conclusions:**

The ROSC and survival after out-of-hospital cardiac arrest varied across EMS agencies and hospitals in Ohio. Top-performing EMS agencies exhibited unique demographic characteristics, used less mechanical CPR, and were more successful in airway placement. These variations in OHCA care and outcomes can indicate opportunities for system improvement in Ohio.

## INTRODUCTION

Nearly 350,000 individuals suffer an out-of-hospital cardiac arrest (OHCA) annually in the United States.^1^ To enhance survival, prehospital recommendations have focused on the importance of 9-1-1 activation, rapid bystander cardiopulmonary resuscitation (CPR), early defibrillation, and provision of high-quality CPR by emergency medical services (EMS).^2^ Despite significant efforts to improve resuscitation performance, survival has remained poor.^3^ Further, regional variability in OHCA outcomes has remained broad.^4^

Studies have described variations in the care and outcomes of life-threatening conditions such as myocardial infarction, stroke, and sepsis.^5^–^7^ While often due to variations in the characteristics of the population,^8^ these differences may also reflect disparities in the organization of care and the skill of care of paramedics and physicians, as well as limitations in institutional resources.^5^ Despite standard professional and community approaches to optimizing OHCA resuscitation care being propagated for over two decades,^2^ significant regional variability in OHCA care persist. 4,9 One study describing variations in OHCA care noted large variations in automated external defibrillator (AED) use and bystander CPR.^9^ Further, national- and state-level evaluations of prehospital OHCA care suggest that rates of survival to hospital admission also vary across EMS agencies.^4^,^10^ These variations in healthcare outcomes may exist within populations that should otherwise be receiving similar care.^5^,^11^,^12^

Surveillance and benchmarking, including identifying high- and low-performing EMS agencies, can potentially identify modifiable factors to optimize OHCA care and outcomes. The Cardiac Arrest Registry to Enhance Survival (CARES) is the nation’s most widespread and durable OHCA registry.^13^ In 2016 Ohio joined CARES as a statewide initiative to improve survival through the registry’s provision for tracking and evaluating care. In this study, we sought to evaluate regional OHCA care variations in the state of Ohio to identify the characteristics that distinguish high-performing EMS agencies and hospitals.

## METHODS

### Study Setting and Design

In accordance with guidelines,^14^ we performed a retrospective analysis of data from Ohio CARES 2019 to evaluate the extent of regional OHCA prehospital care variability. We first evaluated the characteristics of OHCA in Ohio, followed by agency performance variability. Additionally, we evaluated characteristics of top-performing agencies in comparison to average performing agencies. As a national registry of OHCA events, CARES encompasses 2,300 EMS agencies in 46 US states. It obtains data through three resources: 9-1-1 dispatch centers; EMS professionals; and receiving hospitals. This national registry requires that participants achieve >99% data entry and accuracy to be included in the dataset.^15^ In 2019, Ohio participation in CARES included 77 EMS agencies covering 33% of the total state population.^16^ This study was approved by the Ohio CARES Data Sharing Committee and the Ohio State University Office of Responsible Research Practices.

### Study Population

We included all adult (≥18 years) non-traumatic OHCA reported in the 2019 Ohio CARES registry. CARES only includes OHCA with resuscitation efforts, defined as EMS-performed CPR, and/or any defibrillation, including bystander AED use.^13^,^17^ We excluded pediatric OHCA since the underlying etiologies of arrest and quality of resuscitation performance often differ from adults. We also excluded agencies with <10 OHCA episodes in 2019 to ensure a minimum sample size per agency.

**Population Health Research Capsule**

What do we already know about this issue?

*Out-of-hospital cardiac arrest is a leading cause of death. Variations in resuscitation care can contribute to regional differences in outcomes from cardiac arrest.*

What was the research question?

*What are the variations in care and outcomes from cardiac arrest in Ohio?*

What was the major finding of the study?

*Return of Spontaneous circulation varied 3-fold across Ohio with mean 27.9% and range 15.8% to 51.0% of cardiac arrests.*

How does this improve population health?

*Variations in cardiac arrest outcomes can identify opportunities for systems improvements in resuscitation care across Ohio.*

### Outcomes

The primary outcomes were return of spontaneous circulation (ROSC) and survival to hospital discharge determined at the EMS agency and receiving hospital level. We then used these outcomes to define high-performing agencies and hospitals.

### Analysis

First, we described the baseline characteristics of the OHCA in the registry using standard summary statistics. We then compared OHCA outcomes between EMS agencies and receiving hospitals using linear mixed models with agency/receiving hospital as a random effect. We adjusted the model for pertinent covariates, including age, gender, race, witnessed status, bystander CPR, initial rhythm, and location.^18^ Only complete cases were included for modeling, output (Appendix 1). For the comparison across EMS agencies or hospitals, we used a typical cardiac arrest patient: male; White race, age 60 with an unwitnessed cardiac arrest without bystander CPR in a home location.^19^ We defined outliers as EMS agencies or hospitals with 95% confidence interval (CI) performance bands outside the cohort mean.

We used number of cardiac arrests in our cohort to define EMS agency size where median (25 OHCA annually) defined medium-sized agencies, and above the 75^th^ percentile of OHCAs (40 OHCA annually) defined large-sized agencies. We compared EMS agency population demographics, response times, and resuscitation characteristics of the top 10% (rounded to next integer) of agencies outperforming the mean against remaining agencies using chi-squared tests. We performed all analyses using STATA IC version 17 (StataCorp LP, College Station, TX), and ARCGIS (Environmental Systems Research Institute, Redlands, CA).

## RESULTS

**Figure 1:**
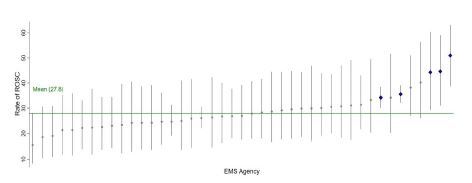
Rate of return of spontaneous circulation (ROSC) across emergency medical service (EMS) agencies. Dots represent individual agency mean ROSC rate with associated standard deviation (bars). ROSC varied 3-fold across EMS agencies with a mean agency average ROSC rate of 27.9%, range 15.8%‒51.0%. Five EMS agencies were top- performing agencies with ROSC rates above the mean EMS agency average (blue dots).

**Figure 2:**
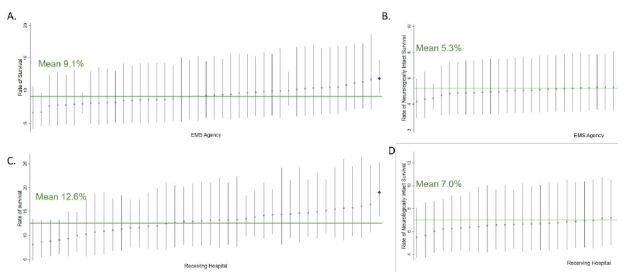
Rate of survival to hospital discharge (left graphs) and neurologically intact survival (right graphs) across emergency medical service (EMS) agencies (top row) and receiving hospitals (bottom row). Dots represent individual agency or hospital mean with associated standard deviation (bars). (A) Survival varied 2-fold across EMS agencies with an agency mean of 9.1%, range 6.6%‒11.9%. One EMS agency had survival rates above the mean (blue dot). (B) Neuro-intact survival varied 1.5-fold across EMS agencies with a mean of 5.3%, range 4.2%‒6.0%. (C) Survival varied 2-fold across receiving hospitals with a mean survival of 12.6%, range 8.1%‒19.0%. One hospital had survival above the mean (blue dot). (D) Neuro-intact survival varied 1.5-fold across receiving hospitals with a mean of neuro-intact survival of 7.0%, range 5.5%‒8.6%.

During 2019, the registry contained 2,991 OHCA treated by 77 EMS agencies. Among 44 included EMS agencies, there were 2,841 OHCAs. Population characteristics were similar to national figures^19^: median age 61 years; male 60.3%; White race 65.9%; witnessed arrests 48.7%; and arrest at home residence 68.9% (Table 1). We determined rates of ROSC and survival to discharge for each EMS agency. Rates of ROSC varied from 15.8%‒51.0% (Figure 1). Five medium-to-large EMS agencies were in the top 10% of performance. No agencies exhibited performance below the mean ROSC rate. The EMS agency rates of OHCA survival to hospital discharge varied from 6.6%‒11.9%. Only one agency outperformed the mean survival rate (9.1%). No agencies exhibited survival below the group mean (Figure 2). Among 40 included receiving hospitals, rates of survival varied from 8.1%‒19.0%. Only one receiving hospital performed above the cohort mean (12.6%). There were no underperforming receiving hospitals. Neurologically intact survival ranged from 5.5%‒8.6%, with no under or over the mean (7.0%) performing hospitals.

We compared characteristics of the top 10% of EMS agencies above the mean. When comparing OHCAs within agencies, top-performing EMS agencies tended to treat younger patients (59 vs 62 years, *P* <0.01) in public areas (15.7% vs 12.3%, *P* <0.01) (Table 2). Other distinguishing characteristics of top-performing EMS agencies included lower utilization of mechanical CPR (61.7% vs 76.0%, *P* <0.01) and higher rates of successful advanced airway placement (89.6% vs 74.8% *P*<0.01). There were no differences in bystander-witnessed arrest (30.5% vs 34.4%), bystander CPR (34.2% vs 36.9%), EMS response time (5 vs 5.1 minutes) (Table 2, Table 3). As there were minimal outliers from the mean and to avoid potential identification, we did not pursue further descriptive statistics for our survival analysis.

## DISCUSSION

The statewide dissemination of CARES data provides the opportunity to compare performance between EMS agencies in their associated communities. In this statewide series from Ohio, we observed three-fold variations in ROSC and two-fold variations in survival to hospital discharge. We were also able to identify high-performing EMS agencies and some of their distinguishing characteristics. We believe these findings illustrate the value of statewide registries for benchmarking OHCA care, because results are more relevant and actionable for state- and agency-level efforts compared to national reports.

Previous studies using CARES to characterize regional resuscitation performance and outcomes have also been performed but differ from the present analysis. Huebinger et al described approximately a two-fold difference in survival across 13 EMS agencies in Texas CARES.^9^ Our sample includes a more diverse range of EMS agencies and higher survival and survival with good neurologic function (survival Ohio 13.1% vs Texas 9.1%; cerebral performance scale 1 or 2 Ohio 8.6% vs Texas 4.0%). Series from North Carolina and Alaska reported higher rates of survival (33.6% and 17.1%, respectively) compared to our study. North Carolina reported similar rates of good neurologic survival, Cerebral Performance Category 1 or 2 (9.7%).^20^

More importantly, the present analysis offers a novel analytic approach toward spotlighting the top-performing EMS agencies in the series to better understand the underlying causes of regional variation. Interestingly, we did not identify any below-average performing agencies, which suggests that variation is driven by the few high-performing agencies in Ohio. Benchmarking EMS agencies is a useful tool that can provide the foundation for community-based OHCA care improvement initiatives for the state rather than targeting individual EMS agency interventions.

Defining site-level variations has motivated practice change and improved outcomes across multiple critical illnesses.^6^,^7^ For example, benchmarking stroke centers has allowed for equitable comparisons to identify modifiable quality improvement strategies.^21^,^22^ Similarly, regional variability in sepsis outcomes highlighted targeted improvement strategies.^23^,^24^ Variations in OHCA outcomes have previously been linked to population, community, and EMS agency factors.^25^–^28^ The chain of survival for cardiac resuscitation focuses on individualized care^29^ without focus on aligning practice patterns across regions. Identification of outcome variations provides opportunities to improve cardiac arrest care through using appropriate targeted community strategies within regions.^30^ For example, the most recent study of variations in EMS resuscitation across the US found that agencies with faster response times were associated with improved survival.^4^ Further, targeted interventions such as improving bystander CPR rates and EMS interventions improved survival.^20^,^31^ Framed with the chain-of-survival ideology, leveraging EMS and community interventions to develop targeted strategies against regional variations, along with additional investigations that identify modifiable factors in high-quality receiving hospitals, can improve survival.

Our findings identified two areas for optimization: manual, high-quality CPR; and successful advanced airway management. Prior studies have shown heterogenous associations with mechanical CPR and advanced airway strategies in resuscitation. Observational studies have shown improved survival with mechanical CPR.^32^–^34^ However, randomized trials and meta-analysis have shown worse neurologic survival with mechanical CPR.^34^–^41^ Similarly, multiple studies have highlighted challenges in advanced airway management and shown both improved and reduced OHCA outcomes compared to bag-mask-ventilation.^42^–^47^ Recent meta-analysis showed improved outcomes with advanced airway strategies.^48^,^49^

Our data revealed an association between manual, high-quality CPR and successful advanced airway placement with improved outcomes. Improvement strategies for advanced airway placement success can include more training, video-assisted laryngoscopy, or potentially placement of supraglottic devices, which has increased in use.^50^,^51^ Despite heterogeneous evidence, advanced airway strategies are used in the majority of OHCA resuscitations, and mechanical CPR utilization has significantly increased in the US.^47^,^52^ Further qualitative studies should evaluate these high-performance agencies to determine whether mechanical CPR use and airway success are the only differences from average agencies.

These observations highlight important opportunities for improving community-wide OHCA care and outcomes through use of statewide registries. Identifying high-quality CPR, one of the initial links in the chain of survival, and ventilation quality metrics are the initial steps in improvement strategies. Validation of these associative quality metrics and outcomes is the next step in implementation. Further, improvement strategies must be applicable to regional barriers and culture. Providing statewide data to pertinent teams, such as statewide CARES teams, can allow for directed and culturally appropriate statewide initiatives to improve OHCA outcomes.^20^,^53^ Targeting improvement strategies toward known weak links in the chain of survival is known to improve OHCA outcomes.^30^,^54^

## LIMITATIONS

This analysis included only an estimated 5.9% of potential EMS agencies in Ohio, which covered 35% of the populus with an urban bias. Inclusion of a larger proportion of the populus and/or more rural agencies could have changed the variation in outcomes and potentially highlighted different factors associated with outcomes. Our study only identified one outlier in both EMS agency and receiving hospital survival. We did not pursue further descriptive statistics for our survival analysis. It is unclear whether directed quality initiatives would result in changes in survival. Lastly, missing data may alter results. The ROSC modeling was able to include >99% of all cases; however, destination hospital was missing in 743 cases, which may have significantly affected distribution predictions. Interventions such as EMS arrival time and start of CPR is not mandatory in CARES reporting. We were missing 2/5 of EMS agency arrival times, which may have altered our findings. We also omitted Agency C EMS CPR initiating time, as values were missing in greater than 50% of cases.

## CONCLUSION

Significant variations in both return of spontaneous circulation and survival exist across EMS agencies in Ohio. Understanding regional variations in prehospital care can provide novel perspectives that can be leveraged to improve care.

## Supplementary Information



## Figures and Tables

**Table 1:** Demographics of out of hospital cardiac arrest in Ohio 2019.

Characteristics	Frequency (%) N=2,841
Age mean (±SD)	61 (±17.2)
Sex n (%)	
Male	1,715 (60.3)
Race n (%)	
White	1,871 (65.9)
Black	829 (28.9)
Other	150 (5.3)
Location of arrest n (%)	
Home/residence	1,982 (68.9)
Nursing home	305 (10.7)
Public/commercial building	219 (7.7)
Healthcare facility	158 (5.7)
Street/highway	135 (4.8)
Industrial place	11 (0.4)
Transport center	1(0)
Witnessed status n (%)	
Unwitnessed	1,458 (51.3)
Bystander witnessed	918 (32.3)
EMS witnessed	465 (16.4)
Initial rhythm n (%)	
Shockable	507(17.9)
Non-shockable	2,334 (82.2)
Bystander CPR n(%)	1,002 (35.3%)
ROSC n (%)	911 (32)
Survival to hospital discharge n (%)	348 (12.3)
Survival with CPC score 1 or 2	242 (8.5)

1=Full recovery or mild disability; 2= Moderate disability but independent in activities of daily living

*EMS*, emergency medical service; *CPR*, cardiopulmonary resuscitation; *CPC*, Cerebral Performance Category; *ROSC*, return of spontaneous circulation.

**Table 2:** Characteristics of out-of-hospital cardiac arrest patients in top 10% of performing EMS agencies in Ohio (A–E).

Agency ID	A	B	C	D	E	Top 10% agencies above mean (combined)	Other performing agencies
Age mean (IQR) *	61 (51–73)	57 (45–70)	66 (62–72)	68 (63–76)	60 (46–72)	59 (48–71)	63 (52–76)
Location (%) *						n (%)	n (%)
Home	65.9	68.9	45.0	69.0	74.5	904 (67.7)	1189 (71.8)
Nursing home/healthcare facility	20.6	13.5	40.0	20.7	12.8	222 (16.6)	262 (15.8)
In general public	13.4	17.5	15.0	10.3	12.8	209 (15.7)	204 (12.3)
Witnessed status (%)						n (%)	n (%)
Unwitnessed	56.5	52.3	35.0	34.5	42.6	706 (52.9)	831 (50.2)
Bystander witnessed	29.1	30.4	25.0	51.7	36.2	407 (30.5)	568 (34.3)
EMS witnessed	14.4	17.2	40.0	13.8	21.3	222 (16.6)	256 (15.5)
CPR initiated by (%)						n (%)	n (%)
EMS	64.9	65.5	55.0	69.0	83.0	879 (65.8)	1045 (63.1)
Bystander	35.1	34.5	45.0	31.0	17.0	455 (34.2)	610 (36.9)
Shockable rhythm (%)						n (%)	n (%)
Yes	21.0	17.8	20.0	34.5	12.8	256 (19.2)	284 (17.2)
No	79.0	82.2	80.0	65.5	87.2	1079 (80.8)	1371 (82.8)
ROSC	34.6	36.6	65.0	62.1	61.7	37.8	32.0
Survival	15.7	11.6	20.0	20.7	19.2	13.6	12.2

* P<0.01

*EMS*, emergency medical service; *IQR*, interquartile range; *CPR*, cardiopulmonary resuscitation; *ROSC*, return of spontaneous circulation.

**Table 3:** Emergency medical services interventions in top 10% performing EMS agencies (A–E).

Agency ID	A	B	C	D	E	Top 10% agencies above mean (combined)	Other performing agencies
EMS arrival time (minutes) mean (IQR)	-	5(3.6–6.1)	3.5(3–4.5)	4.3(3–5.1)	-	5.0(4.0–6.0)	5.1(4.9–5.4)
EMS time to CPR minutes mean (IQR)	-	0.8(0–2)	-	1.1(0–2)	-	1(0–2.9)	1.2(0–3)
Mechanical CPR (% yes)*	46.4	69.9	60.0	65.5	84.7	61.7	76.0
Advanced airway successfully placed (% yes) *	89.6	78.5	80	65.5	84.8	89.6	74.8
Supraglottic airway	32.3	35.6	0	0	2.4	32.3	30.9
Endotracheal tube	67.6	64.4	100	100	97.6	67.7	69.0

“-” = missing greater than 50% of values,

* P<0.01.

*EMS*, emergency medical services; *IQR*, interquartile range; *CPR*, cardiopulmonary resuscitation.
