# Supplementary material for: Variations in Out-of-Hospital Cardiac Arrest Resuscitation Performance and Outcomes in Ohio
Source: West J Emerg Med. 2025 Mar 15;26(3):541–8. doi: 10.5811/westjem.19422 (PMC12208045; doi:10.5811/westjem.19422)
Supplement: Supplementary file 1 [file wjem-26-541-s001.docx]

| Covariates | OR (95% CI) |
| --- | --- |
| Age | 1.0 (0.99-1.00) |
| Male Gender | 1.28 (1.08-1.52) |
| Witnessed Arrest | 2.51 (2.11-2.99) |
| Bystander CPR | 1.11 (0.91-1.34) |
| Race  White  Black or African-American  Other/Unknown | Ref  0.87 (0.72-1.07)  1.25 (0.87-1.81) |
| Shockable Rhythm | 1.56 (1.26-1.93) |
| Location  Residence or Home  Nursing Home or Health Care Facility  General Public | Ref  0.93 (0.71-1.20)  1.11 (0.88-1.43) |

**Appendix Table 1: Mixed Effects Model for ROSC** EMS agency was used as a random effect. Area Under the Curve =0.65 with Hosmer-Lemeshow Testing p=0.57
